# Genomic Characterization Helps Dissecting an Outbreak of Listeriosis in Northern Italy

**DOI:** 10.1371/currents.outbreaks.633fd8994e9f06f31b3494567c7e504c

**Published:** 2017-07-06

**Authors:** Francesco Comandatore, Marta Corbella, Giuseppina Andreoli, Erika Scaltriti, Massimo Aguzzi, Stefano Gaiarsa, Bianca Mariani, Marina Morganti, Claudio Bandi, Massimo Fabbi, Piero Marone, Stefano Pongolini, Davide Sassera

**Affiliations:** S.C. Microbiologia e Virologia, Fondazione IRCCS Policlinico San Matteo, Pavia, Italia; Istituto Zooprofilattico Sperimentale della Lombardia e dell’Emilia Romagna, Pavia, Italy; Servizio di Analisi del Rischio, Direzione Sanitaria, Istituto Zooprofilattico Sperimentale della Lombardia e dell'Emilia-Romagna (IZSLER), Parma, Italy; Dipartimento di Prevenzione Veterinaria, Agenzia della Salute di Pavia, Pavia, Italy; SC di Microbiologia e Virologia, Fondazione IRCCS Policlinico San Matteo, Pavia, Italy; S.C. Microbiologia e Virologia, Fondazione IRCCS Policlinico San Matteo, Pavia, Italia; Servizio di Analisi del Rischio, Direzione Sanitaria, Istituto Zooprofilattico Sperimentale della Lombardia e dell'Emilia-Romagna (IZSLER), Parma, Italy; Dipartimento di Bioscienze, Università degli Studi di Milano, Milano, Italy; Istituto Zooprofilattico Sperimentale della Lombardia e dell'Emilia Romagna, Pavia, Italy; SC Microbiologia e Virologia, Fondazione IRCCS Policlinico San Matteo, Pavia, Italy; Direzione Sanitaria, Servizio di Analisi del Rischio, Istituto Zooprofilattico Sperimentale della Lombardia e dell'Emilia-Romagna (IZSLER), Parma Italy

## Abstract

**Introduction:**

Listeria monocytogenes (Lm) is a bacterium widely distributed in nature and able to contaminate food processing environments, including those of dairy products. Lm is a primary public health issue, due to the very low infectious dose and the ability to produce severe outcomes, in particular in elderly, newborns, pregnant women and immunocompromised patients.

**Methods:**

In the period between April and July 2015, an increased number of cases of listeriosis was observed in the area of Pavia, Northern Italy. An epidemiological investigation identified a cheesemaking small organic farm as the possible origin of the outbreak. In this work we present the results of the retrospective epidemiological study that we performed using molecular biology and genomic epidemiology methods. The strains sampled from patients and those from the target farm's cheese were analyzed using PFGE and whole genome sequencing (WGS) based methods. The performed WGS based analyses included: a) in-silico MLST typing; b) SNPs calling and genetic distance evaluation; c) determination of the resistance and virulence genes profiles; d) SNPs based phylogenetic reconstruction.

**Results:**

Three of the patient strains and all the cheese strains resulted to belong to the same phylogenetic cluster, in Sequence Type 29. A further accurate SNPs analysis revealed that two of the three patient strains and all the cheese strains were highly similar (0.8 SNPs of average distance) and exhibited a higer distance from the third patient isolate (9.4 SNPs of average distance).

**Discussion:**

Despite the global agreement among the results of the PFGE and WGS epidemiological studies, the latter approach agree with epidemiological data in indicating that one the patient strains could have originated from a different source. This result highlights that WGS methods can allow to better

## Competing Interests

I, Davide Sassera, on behalf of all the authors of the manuscript ‘Genomic characterization helps dissecting an outbreak of Listeriosis in Northern Italy’ submitted to Plos Current Outbreaks DECLARE that no competing interests exist.

## Data Availability Statement

I, Davide Sassera, on behalf of all the authors of the manuscript ‘Genomic characterization helps dissecting an outbreak of Listeriosis in Northern Italy’ submitted to Plos Current Outbreaks [OBK-17-0002] declare that the minimal dataset underlying the findings in the manuscript is publicly available to other researchers. The data were submitted to the European nucleotide database (ENA), which is the database of the European Bioinformatics Institute (EBI) which belongs to the European Molecular Biology Laboratory (EMBL). Specifically, the sequencing reads are made available under the accession number PRJEB20581 and the genome assemblies are made available under the accession numbers ERS1607070-ERS1607078.

## Corresponding Author

The corresponding author is davide sassera, davide.sassera@unipv.it.

## Introduction

*Listeria monocytogenes* (Lm), widely distributed in the environment including soil, plants, and water, is a foodborne bacterial pathogen that can contaminate different kinds of food among which milk and dairy products^1^. Lm is capable of adapting to and growing at refrigeration temperatures and, moreover, it can form biofilm to help colonization of surfaces. Consequently, Lm can colonize food processing environments, contaminating the finished products^2^. Although Lm is an uncommon cause of illness in the general population, it can represent an important public health problem in case of large scale distribution of contaminated food, due to the very low infectious dose^3^^**,**^^4^. Listeriosis is a severe disease and it primarily affects the elderly 5, newborns, pregnant women and immunocompromised patients, categories that can be up to 20 times more susceptible to the disease^6^. Clinical manifestations are highly variable and host-dependent: from non-specific and mild symptoms, to febrile gastroenteric syndromes or even cases of seps is and meningitis with mortality rates up to 30%^7^.

Most reported cases of listeriosis are sporadic, however, outbreaks have been described with increasing incidence worldwide^5^^,^^8^^,^^9^^,^^10^^,^^11^. ECDC and EFSA report 2,161 confirmed human cases of listeriosis in the EU Summary report on zoonoses, zoonotic agents and food-borne outbreaks 2014^12^. The EU notification rate was 0.52 cases per 100,000 population which represents a 30% increase compared with 2013 (0.40 cases per 100,000 population). ECDC and EFSA report 210 deaths due to listeriosis in 2014, and a fatality rate above 12.5%. This was the highest number of deaths reported since 2009 (annual average: 163). An average of 131 cases with 0.22 cases per 100,000 population were reported in Italy from 2010 to 2013^12^. A regional study regarding the Lombardia region reported 134 isolates in the 2006-2010 period^6^. The notification of listeriosis in humans is mandatory in most countries in Europe, in Italy since 1991, as regulated by Italian D.M. 15/12/1990. Since 2009, was established a digital platform, ENTER-NET Italia system (Enteric Pathogen Network) connected to European ENTER-NET network, dedicated to the assessment of microbiological clusters of food-borne diseases^13^.

Pulsed-Field Gel Electrophoresis (PFGE) represents the gold standard for subtyping of Lm and other foodborne pathogens^14^, however, studies employing other molecular and genomic methods have proliferated recently, allowing to characterize Lm strains not only by pulsotype, but also by multilocus sequence type (MLST) and core genome MLST (cgMLST) 15^,^^16^^,^^17^^,^^18^****.

In this study we describe an outbreak of *Listeria monocytogenes* occurred in 2015 in Northern Italy, using a combination of molecular biology and genomics techniques. Despite the results obtained from the two approaches resulted coherent at large scale, the Whole Genome Sequencing (WGS) approach resulted more accurate in the discrimination of the strains involved in the outbreak.

## The outbreak

Between 28th April and 11th July 2015 six patients showing symptoms compatible with Listeriosis (sudden onset of fever, chills, severe headache, vomiting, and other influenza-like symptoms) were admitted to hospitals in Pavia province of Lombardia region, Northern Italy. The first three cases were observed at the Fondazione IRCCS Policlinico San Matteo Hospital in Pavia (Italy), the fourth an the Ospedale SS Annunziata di Varzi, the fifth an the Ospedale unificato di Broni-Stradella and the sixt at the Ospedale Civile di Voghera. We will refer to the patients enumerating them chronologically from 1 to 6 (Table 1).

**Table 1:** Characteristics of the patients and of the cheese samples where *Listeria monocytogenes *was detected.

Sample name	Geographic origin	Sampling date	Source	Age	Gender	Risk factor	Diagnosis and symptoms	Outcome
Patient_1	Pavia	2015/04/28	blood and CSF	71	M	no one	Meningitis and sepsis	cured
Patient_2	Pavia	2015/04/29	blood	47	M	HIV and HCV infection, hepatic impairment	HIV, HCV and HBV co-infection, jaudice, sepsis	cured
Patient_3	Pavia	2015/05/14	CSF	1.5	M	< 2 year aged	Diarrhea, fever and confusion	hydrocephalus
Patient_4	Varzi	2015/05/21	blood	78	M	no one	Sepsis	cured
Patient_5	Broni-Stradella	2015/07/11	blood	61	F	cancer	Sepsis	death
Patient_6	Voghera	2015/07/28	blood	70	M	Parkinson disease	Diarrhea, Meningitis	cured
Cheese_1	Target farm	2015/05/26	Cheese (inner)	na	na	na	na	na
Cheese_2	Target farm	2015/05/26	Cheese (rind)	na	na	na	na	na
Cheese_3	Target farm	2015/07/17	Cheese (rind)	na	na	na	na	na

Patient 1, upon admission, informed the hospital personnel of having recently consumed goat cheese produced by a small organic farm. The patient provided the leftover cheese (~ 20g), that was tested and Lm was not detected. It must be noted that the small amount of available cheese could have influenced the sensitivity of the test, as the standard protocol indicates 25g as the correct amount for the analysis.

During the following two weeks, two apparently unrelated listeriosis cases were observed at Pavia hospital, involving a drug-abusing subject (patient 2) and a 1.5 year old child (patient 3). The child’s parents informed the hospital personnel that the child had recently consumed home-made cheese, and provided a cheese sample (>25g) which was tested, and no Lm was detected. During the previous three years an average of 3.3 cases per year were observed in Pavia province, thus, three cases in 16 days were considered a possible outbreak, and an epidemiological investigation was performed.

The farm that produced the raw-milk goat cheese eaten by patient 1 was subjected to a first inspection during which goat cheese and raw milk, as well as the food contact surfaces in the processing plant^19^ were sampled. Lm was isolated from two samples from a single cheese shape (Cheese_1 and Cheese_2 in Table 1), while one of three samples collected from the plant (wood ripening surface) was found to be positive by PCR. In order to monitor the possible persistence of Lm contamination three additional sampling were performed during the two months after the first inspection. One wood ripening surface resulted positive by PCR in June, and Lm was isolated from a cheese shape in July. (Cheese_3 in Table 1). All samples collected in the farm are showed in Table 2.

**Table 2:** Samples from the putative target farm. Numbers indicate collected samples / PCR positive samples / isolation positive samples

Collection date	Cheese samples	Milk samples	Food-contact surface samples	Non food-contact surface samples
2015/05/22	2/2/2		3/1/0	
2015/05/15		1/0/0		
2015/06/19	2/0/0		6/1/0	4/0/0
2015/07/17	4/1/1		5/0/0	2/0/0
2015/09/23			7/0/0	8/0/0

During the epidemiological investigation three other cases of listeriosis were diagnosed in the province of Pavia. None of the three patients reported to have eaten organic cheese, or other useful information for investigators to trace the origin of the infections. Four patients out of the six completely recovered, while patient 3 developed a hydrocephalus and patient 5 died. All the isolates were subjected to molecular characterization and, subsequently, a retrospective genomic investigation was performed.

## Methods

Ethics statement

The study was designed and conducted in accordance with the Helsinki declaration. This study was performed according to the guidelines of the IRCCS Foundation Policlinico San Matteo Hospital in Pavia Institutional Review Board of on the use of biological specimens for scientific purposes in keeping with Italian law (art.13 D.Lgs 196/2003). The work described here is a retrospective study performed on bacterial isolates from human samples that were obtained as part of hospital routine. No extra human samples were obtained for this research. Therefore, informed consent (either written or verbal) was not required.

Strain isolation

Blood and Cerebrospinal Fluid (CSF) samples obtained from the six patients were inoculated in aerobic or pediatric broth, and incubated in BACTEC FX (Becton Dickinson, Heidelberg, Germany). Positive broths from blood and CSF were analyzed by Gram staining method and culture, matrix-assisted laser desorption/ionization time-of-flight (MALDI-TOF) mass spectrometry (MS) MicroflexTM LT (Bruker Daltonik GmbH, Bremen, Germany) was used for species identification through the Bruker biotyper 3.1 database. Antibiotic susceptibility tests of each isolate was performed via standard disk diffusion on Mueller-Hinton agar incubated at 37°C for 24 h using the Kirby-Bauer method^20^. The results were interpreted with standardized criteria from breakpoint committee EUCAST^21^. All isolates were then stocked at -80° C. The cheese samples provided by patient 1 and 3 were subjected in parallel to molecular diagnosis and isolation protocols, respectively PCR Real Time – iQCheckTM *L. monocytogenes* II kit (BIORAD) AFNOR BRD 07/10 – 04/05 and ISO 11290-1:116/Amd 1:2004^22^. eight cheese, one raw milk and 35 environmental samples were collected from the putative origin dairy processing plant. All these samples were subjected to the above PCR method and PCR positive samples were subjected to standard Lm isolation protocol, according to ISO 11290-1:1996/Amd 1:2004^22^**.**

Molecular characterization

All isolates were subjected to DNA extraction using the Qiagen DNeasy kit according to manufacturer's instructions, and to Lm specific PCR using an accredited protocol (PCR real-time - iQ-CheckTM *L. monocytogenes* II kit (BIO-RAD) AFNOR BRD 07/10 – 04/05). All isolates were genotyped by PFGE according to the Pulsenet protocol^23^. Genomic DNA underwent restriction with *AscI* and *ApaI* enzymes before electrophoresis in a CHEF Mapper® XA System (Bio-Rad, California, USA). PFGE patterns were analyzed using Bionumerics Software ver. 7.0 (Applied-Maths, Sint-Martens-Latem, Belgium) and associated to strain information in our surveillance database. Clustering of the PFGE profiles was generated using the Unweighted Paired Group Method with arithmetic averages (UPGMA) based on the Dice Similarity Index (Optimization=1% and Band Matching Tolerance=1%). Following comparison of the electrophoretic profiles, a PFGE pattern (pulsotype) was assigned to each isolate within the database of the laboratory of the Istituto Zooprofilattico Sperimentale della Lombardia e dell’Emilia Romagna (Sezione Diagnostica di Parma). Two isolates were indicated as belonging to the same pulsotype if the band pattern differed by less than two bands.

Genomics

Whole-genome DNA was extracted from each isolate using a QIAamp DNA minikit (Qiagen) following the manufacturer's instructions, and sequenced using an Illumina Miseq platform with a 2 by 250 paired-end run after Nextera XT paired-end library preparation. Genome assemblies were obtained using Mira software^24^ and subjected to open reading frame (ORF) calling using Prodigal^25^. The MLST profiles of the sequenced strains were determined in silico (using an in-house Perl script), on the basis of the MLST profiles defined in the Institut Pasteur MLST database (http://bigsdb.pasteur.fr/listeria/26). The 713* Lm *genomes available in the Patric database (in date 11th July 2016) were retrieved and subjected to in silico MLST profile determination and the genomes belonging to the same clonal complexes of our strains were selected for further analyses. The selected strains were subjected to core genome SNP-based phylogeny. The analysis was performed on a robust dataset of core genes selected from the cgMLST1748 genes^27^****. The cgMLST1748 genes were extracted from BIGSdb-Lm platform and searched, using Blastn, in the genomes of the selected strains. Bidirectional Best Hit (BBH) method^28^ **** was then used to group the genes into clusters of orthologous genes. For each cgMLST1748-ortholog gene present in single copy in all the genomes, the sequences of all isolates were retrieved, aligned and translated using in-house Perl scripts and Muscle software^29^. The cgMLST gene alignments were then screened and the genes with the following features were selected: a) all the aligned sequences begin with a start codon; b) all the aligned sequences finish with a stop codon; c) all the aligned sequences have a single stop codon; d) for each aligned sequence, the gaps cover less than 10% of the alignment length. The nucleotide alignments of the selected cgMLST1748 orthologs were then concatenated and subjected to phylogenetic analysis using Maximum Likelihood approach, with RaxML 8 software^30^, setting GTRGAMMA model and 100 pseudo bootstrap replicates.

Four databases of Lm sequences, namely virulence genes, antibiotic resistance genes, genes for resistance to Benzalkonium and genes for resistance to metals and detergents were retrieved from the ListeriaMLST database. For each genome sequenced in this study, the obtained paired-end reads were aligned against the curated genes databases using Bowtie2. Genes with >10X coverage for >95% of the sequence length were considered as present in the isolate.The genomes were searched for presence of phages using Phast^31^.

## Results

Case characteristics

The median age of the six patients involved in the study was 54 years (range 1-78), five out of the six were males and all of them lived in the Pavia province. In four out of six patients Lm was isolated from blood cultures, in one patient from cerebrospinal fluid (CSF) and in another one from both blood and CSF. For full details on patients and symptoms see Table 1. An epidemiological investigation identified the cheesemaking small organic farm that possibly originated the outbreak, where sampling of milk, cheese and food processing environment was performed. Lm isolation was achieved from two cheese shapes, in the first case from both crust and paste, in the second case from the crust only. PCR positivity was obtained for 2 farm environment samples.

Isolate characterization

All isolates were susceptible to ampicillin, erythromicin, meropenem, cotrimoxazole, penicillin. The result of the clustering analysis based on the PFGE patterns obtained with *ApaI* and *AscI* enzymes resulted congruent, grouping the isolates collected from patients 1, 2, 4, together with those from the three cheese samples in both analyses, indicating a clear relationship between the six. Isolates from the three other patients showed a clearly different PFGE pattern, excluding their belonging to the outbreak (Figure 1).

**PFGE profiles:**
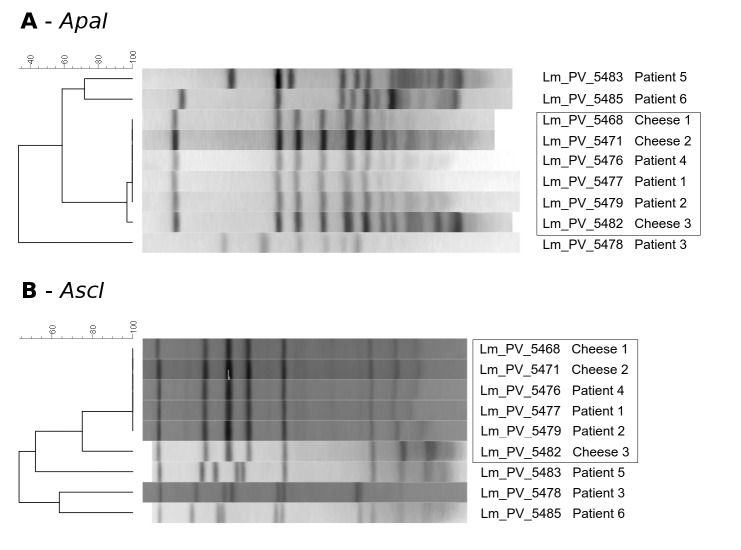
Pulse Field Gel Electrophoresis analysis of the nine strains, obtained using the AscI and ApaI restriction enzymes

Whole genome sequencing analysis

Whole genome sequencing was performed for the nine strains, six from patients and three from cheese. Genome assemblies, submitted to the EMBL-EBI database, resulted to be on average of high quality (Table 3). In-silico MLST was performed on the genome assemblies, revealing that the three isolates obtained from the cheese samples and three of the six isolates from patients belong to sequence type 29 (ST29), and the remaining three isolates belong to ST1, ST7 and the ST398 (see Table 3 for genome statistics and STs).

**Table 3**

**Figure:**
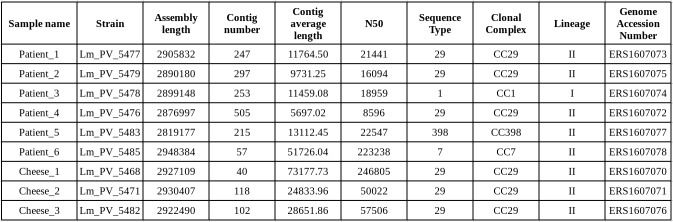
Statistics of the genomes assemblies obtained from nine Listeria monocytogenes strains and MLST profiles.

The 713 Lm genomes present in the Patric database were retrieved, in-silico MLST typed, and the 81 genomes belonging to the clonal complexes of the study strains (i.e. CC1, CC7, CC29 or CC398) were selected. A cgMLST-based phylogenetic reconstruction was performed using a subset of the cgMLST1748 scheme genes, including only the 928 genes present in single copy in all the strains and giving a good quality alignment. The cgMLST-based phylogeny shows that the isolates from patient 1, patient 2, patient 4, cheese 1, cheese 2 and cheese 3 are tightly related (Figure 2), while the other isolates are scattered on the tree. The six closely related strains were then investigated more in depth in order to reconstruct the outbreak structure, using whole genome sequencing (WGS) typing, and data from the epidemiological investigation. In particular, the following evidence was considered:

a) Single nucleotide polymorphism (SNP) distance revealed that the strains from patient 1, patient 2, cheese 1, cheese 2 and cheese 3 differ by 0.8 SNPs on average (values ranging from 0 to 2), while their average distance from patient 4 isolate is ten times higher, at 9.4 SNPs (values ranging from 9 to 10) (see Figure 3).

b) Patient 1 reported to have eaten the cheese produced by the suspect farm, while patient 4, referred to have never bought cheese from the farm. No information on whether patient 2 ate the cheese became available. A potential, albeit unlikely, link would be that patient 4 could have eaten foods prepared with raw materials in common with the contaminated cheese, such as salt solution.

The combination of the higher SNP distance, and the absence of an epidemiological link, led us to consider patient 4 as not associated to the outbreak.

**Phylogeny:**
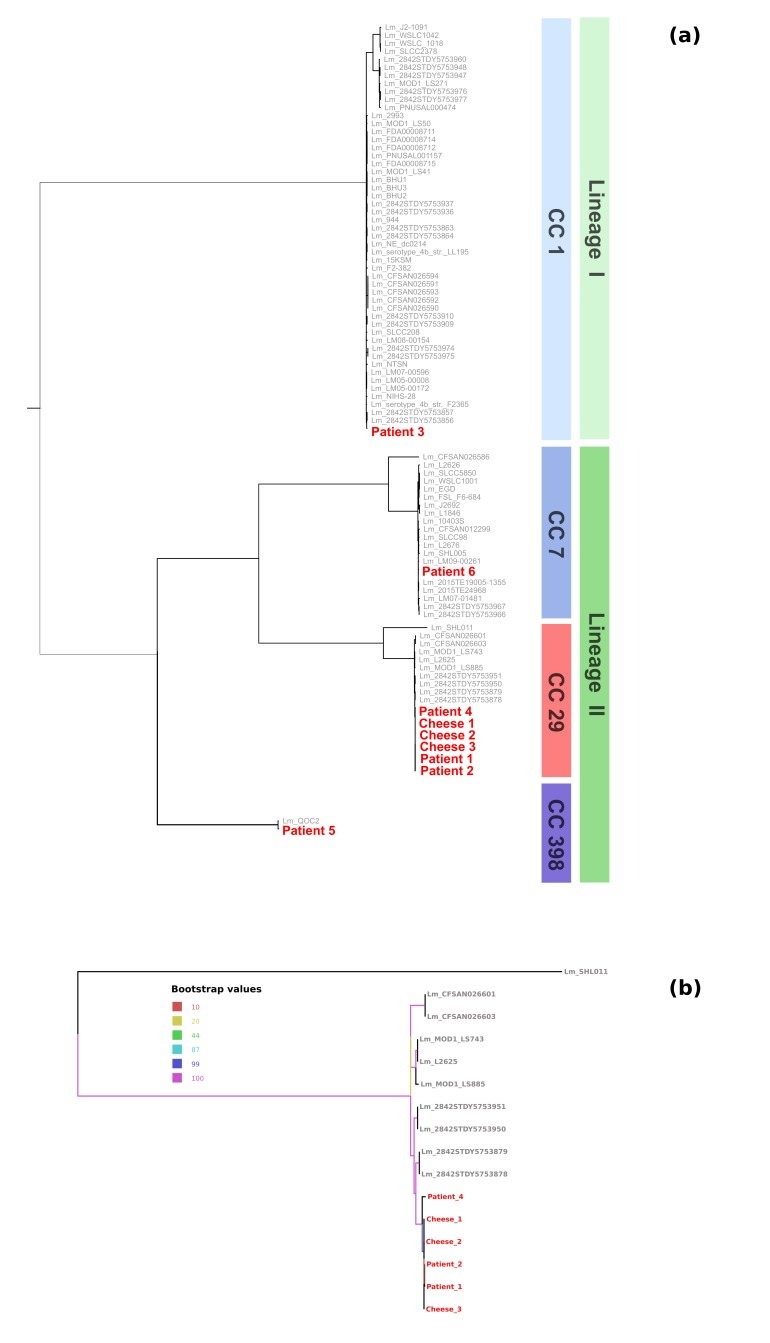
(a) Phylogenetic reconstruction of the relationships between the study isolates and database isolates of the corresponding clonal complexes. Tree obtained using Maximum Likelihood approach, with RAxML 8 software, setting GTRGAMMA model and 100 pseudo bootstrap replicates on an alignment of 928 conserved core genome MLST genes. (b) Sub-tree including only the CC29 strains.

Regarding the presence of resistance genes, all the strains collected in this study showed the same profile of antibiotic resistance genes, harboring the *fos*X, *lmo*1708, *nor*B, and sul genes. This genetic uniformity is in accordance with the results obtained in the antibiograms, which were identical for all strains. Conversely, the virulence genes profiles resulted less conserved among the lineages: the isolates belonging to ST29 (collected from patient 1, patient 2, patient 4, cheese 1, cheese 2, cheese 3 samples) and ST7 (patient 6) presented the same virulence gene profile, while the isolate from patient 5 (ST398) also possessed the vip gene. The isolate from patient 3 (ST1) had multiple additional virulence genes: *aut *IVb, *glt*A, *glt*B, *mdr*M, *vip* and genes of the cluster LIPI-3 (Figure 4). This gene cluster have been reported in the literature to be one of the three major virulence factors (LIPI-1, LIPI-2, LIPI-3)^3^. Seven phages were detected, showing an identical pattern of presence/absence in all the strains belonging to ST29. See Figure 4 for a list of the detected phages.

**Heatmap of the SNP distances:**
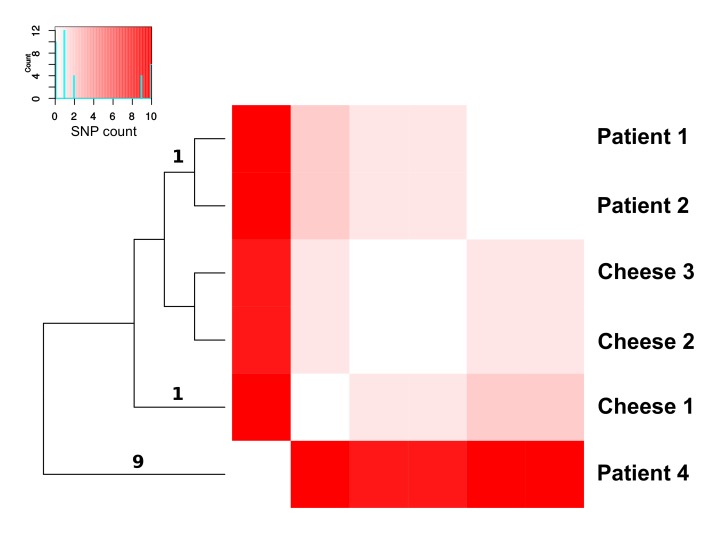
Heatmap showing the single nucleotide polymorphism between the isolates obtained in this study. Bright red corresponds to the highest number of SNPs. The number of SNPs supporting tree branched are reported on the relative branch

**Presence/absence of resistance genes, virulence genes and phages:**
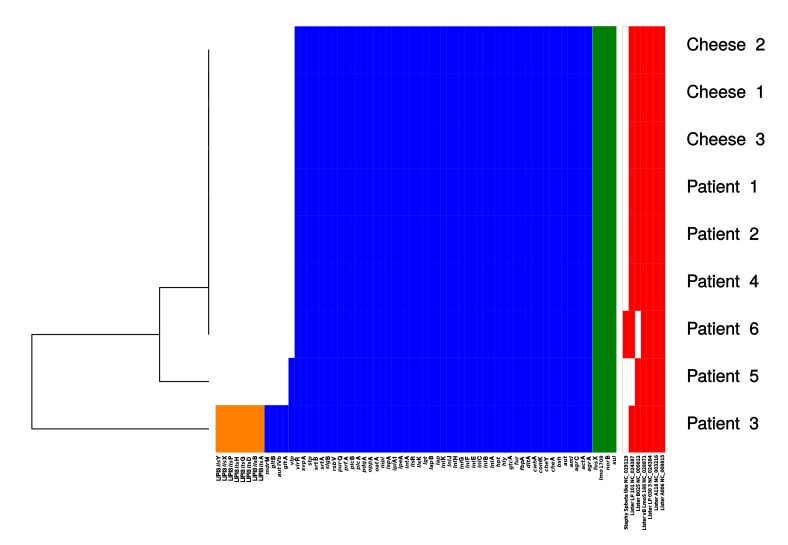
Profiles of presence of genes of interest, including genes for antibiotic resistance and virulence, and phages for each genome. LIPI genes are reported in orange, virulence genes in blue, resistance genes in green and phages in red.

## Discussion

Six cases of listeriosis occurred between 28th April 2015 and 28th July 2015 in four hospitals of the province of Pavia, Northern Italy. This represented an important increase of the incidence in the area, from an average of 0.28 per month in the three previous years to 2 per month in the examined period. This suggested that an *Lm *strain could be emerging in the area, and an epidemiological investigation was performed. In particular, a first investigation was carried out using molecular techniques and patient interviews, and, after one year, a WGS investigation followed. The results of the two reconstructions were then compared.

PFGE clustered together the strains from three patients (patient 1, 2 and 4) and from all the cheese samples collected from the farm identified as the outbreak origin, indicating that the outbreaking strain originated from that farm and then infected the three patients. Patients 3, 5, and 6 resulted to be unrelated to the outbreak. Additionally, the farm environment was *Lm* positive by PCR, prompting the owner to refurbish the structure. The main inconsistency of this reconstruction was patient 4, who declared with confidence to have never eaten the cheese produced at the farm, while he stated to have consumed raw meat, but could not indicate the origin.

The results of the retrospective WGS investigation allowed to better investigate this point. The core genes SNPcgMLST phylogenetic reconstruction clustered the strains from patient 1, 2, 4 and the cheese strains together, in accordance with the PFGE clustering. We then calculated the number of SNPs between each pair of strains of the PFGE cluster. This analysis showed that the isolate from patient 4 presents an average SNP distance ten times higher than the average distance within the cluster (Figure 3). This pattern suggests that patient 4 could had been not part of the outbreak but, having a sole outlier strain, it was not possible to statistically test this hypothesis. Despite this, epidemiological data resulted coherent to the scenario we inferred from WGS data: since these six strains were collected in a span of three months, and the isolate from patient 4 was obtained in the middle of this period, such difference is unlikely to have arisen from multiple mutations of the isolate from patient 4.

On the basis of the collected data we propose the following epidemiological scenario: patient 1 and 2 were infected by the cheese from the target farm, while patient 4 acquired the bacterium from an unidentified source. Furthermore, we suggest that a WGS-based surveillance program could allow to detect this unidentified source, and solve similar cases in the future.

In summary, WGS allowed to characterize the six human isolates of Lm showing that they represent five different clonal clades that circulate in the studied area, all belonging to STs that were previously reported in the region^6^. ST 29 is commonly described in *Lm* outbreaks in USA and Europe, and it was previously described as capable of causing invasive illness^32^^,^^33^. Two closely related clones, both belonging to ST29, were discriminated through genomics leading to accurate assignment of cases to the outbreak source. The close relatedness of the two clones in absence of a demonstrated epidemiological link opens a question about their possible common ancestry and its associated shared environmental niche. Prospective genomic epidemiology investigations focused on ST29 in the area could allow to understand whether these clones are still circulating in the human population and potentially find clues about their environmental niche.
